# UAS-based tracking of the Santiaguito Lava Dome, Guatemala

**DOI:** 10.1038/s41598-020-65386-2

**Published:** 2020-05-25

**Authors:** Edgar U. Zorn, Thomas R. Walter, Jeffrey B. Johnson, René Mania

**Affiliations:** 1 German Research Centre for Geosciences GFZ, Telegrafenberg, 14473 Potsdam, Germany; 2 Boise State University, Department of Geosciences, Boise, ID United States

**Keywords:** Natural hazards, Geology, Volcanology

## Abstract

Imaging growing lava domes has remained a great challenge in volcanology due to their inaccessibility and the severe hazard of collapse or explosion. Changes in surface movement, temperature, or lava viscosity are considered crucial data for hazard assessments at active lava domes and thus valuable study targets. Here, we present results from a series of repeated survey flights with both optical and thermal cameras at the Caliente lava dome, part of the Santiaguito complex at Santa Maria volcano, Guatemala, using an Unoccupied Aircraft System (UAS) to create topography data and orthophotos of the lava dome. This enabled us to track pixel-offsets and delineate the 2D displacement field, strain components, extrusion rate, and apparent lava viscosity. We find that the lava dome displays motions on two separate timescales, (i) slow radial expansion and growth of the dome and (ii) a narrow and fast-moving lava extrusion. Both processes also produced distinctive fracture sets detectable with surface motion, and high strain zones associated with thermal anomalies. Our results highlight that motion patterns at lava domes control the structural and thermal architecture, and different timescales should be considered to better characterize surface motions during dome growth to improve the assessment of volcanic hazards.

## Introduction

Lava domes are among the most hazardous and unpredictable volcanic features, making safe observations and studies challenging for researchers. They form when the extruding lava is too viscous to flow far and thus piles up into a mound-shaped dome, typically steep sided with a flat top^1,2^, although more complex morphologies can develop depending on extrusion rates and lava rheology^3,4^ as well as resulting endogenous or exogenous growth styles^5^. Episodes of lava dome growth have occurred at over 120 volcanoes since 1000 AD, sometimes lasting several decades^6,7^. The many-faceted interactions between extrusion and lava degassing, cooling and crystallization can cause pressurization and gravitational instability in these systems^8^. Therefore, lava domes potentially produce sudden explosions or dome collapses. These are also the origin of destructive pyroclastic density currents, the volcanic hazard responsible for most fatalities during eruptions worldwide, with single events sometimes causing thousands of deaths^9^, highlighting the need for accurate hazard assessments at growing lava domes.

To understand the growth processes and hazards associated with lava domes, critical data such as lava viscosity, dome morphology, growth rate, strain and strain rates, or surface temperature are required. Remote sensing observations on the growth patterns, commonly made by optical^10^, radar^10–13^, and thermal^10,14,15^ satellite sensors as well as ground-based^12,16,17^ or aerial^2,18,19^ photogrammetry, can reveal the morphology, size and growth rate of lava domes as well as indicate the direction of growth and most likely the hazards^19^. Similarly, insight on lava properties, such as viscosity, can provide valuable information on the hazard state, as they can control the explosive behaviour of a lava dome^8^. Lava viscosity data is particularly challenging to acquire because *in-situ* measurements are normally impossible, due to the high hazard or inaccessibility, and are instead retrospectively inferred point-wise by testing selected samples after eruptive activity has ceased^20,21^. Indirect methods of viscosity assessment are possible^1,22,23^, but limited to ground based imaging. Another important aspect in assessing dome hazards are surface temperature measurements. These can be used to detect thermal anomalies on lava domes and reveal structures associated with deformation. A common structural feature is a ring-shaped anomaly observed around the flat top of a lava dome^24,25^. Although attempts to link such thermal anomalies to dome deformation have been made previously, the data resolution did not allow for conclusive statements^26^. Thermal imaging can also improve surface observations in low visibility due to degassing, cloudy conditions, or bad lighting^27^. Surface strain measurements during lava dome growth are rarely considered, but have been previously derived from fixed camera images^28^ and can assist in identifying structural features. Despite these advances, detailed insights on the growth styles of lava domes, such as internal growth (endogenous) and growth by extrusion of lava (exogenous), are limited and our understanding on the timescales involved are incomplete.

To overcome some of these challenges, Unoccupied Aircraft Systems (UASs) have been increasingly used in the observation of volcanic activity^29,30^. This is due to their increased ease of use, refinements in flight technology, capacity to carry large sensors, and improvements in flight durations and distances. This has opened many new possibilities in gathering and interpreting relevant volcanological data. Volcanic areas of varying sizes can be surveyed with relative ease, and Structure-from-Motion (SfM) photogrammetry allows for the creation of detailed 3D models and high resolution digital elevation models (DEMs), which may facilitate the identification of cm-scale features of lava flows and other volcanic surfaces^31^, as well as precise eruptive volumes^32^. Similarly, UAS surveys have been used to map and measure the topographic changes in active volcanic settings^33^. On a few lava domes, the high resolution capabilities of UASs have allowed for highly detailed insights into the structure of the fracture network^34^. Another UAS study conducted repeated surveys with thermal sensors on board that facilitated the assessment of the thermal inertia of a lava flow^35^.

**Figure 1:**
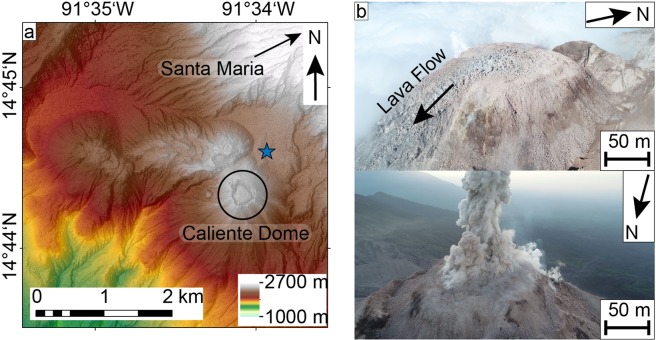
(**a**) Local DEM map of the study area built from Pléiades satellite data, the blue star marks the starting point of our UAS surveys. (**b**) Aerial photos of the Caliente lava dome showing the lava flow (upper photo, taken 18.02.2019 at 15:17 UTC) and a low intensity explosion (lower photo, taken 15.02.2019 at 15:18 UTC). (a) was plotted in ArcMap (v10.5, https://desktop.arcgis.com/de/arcmap/).

**Figure 2:**
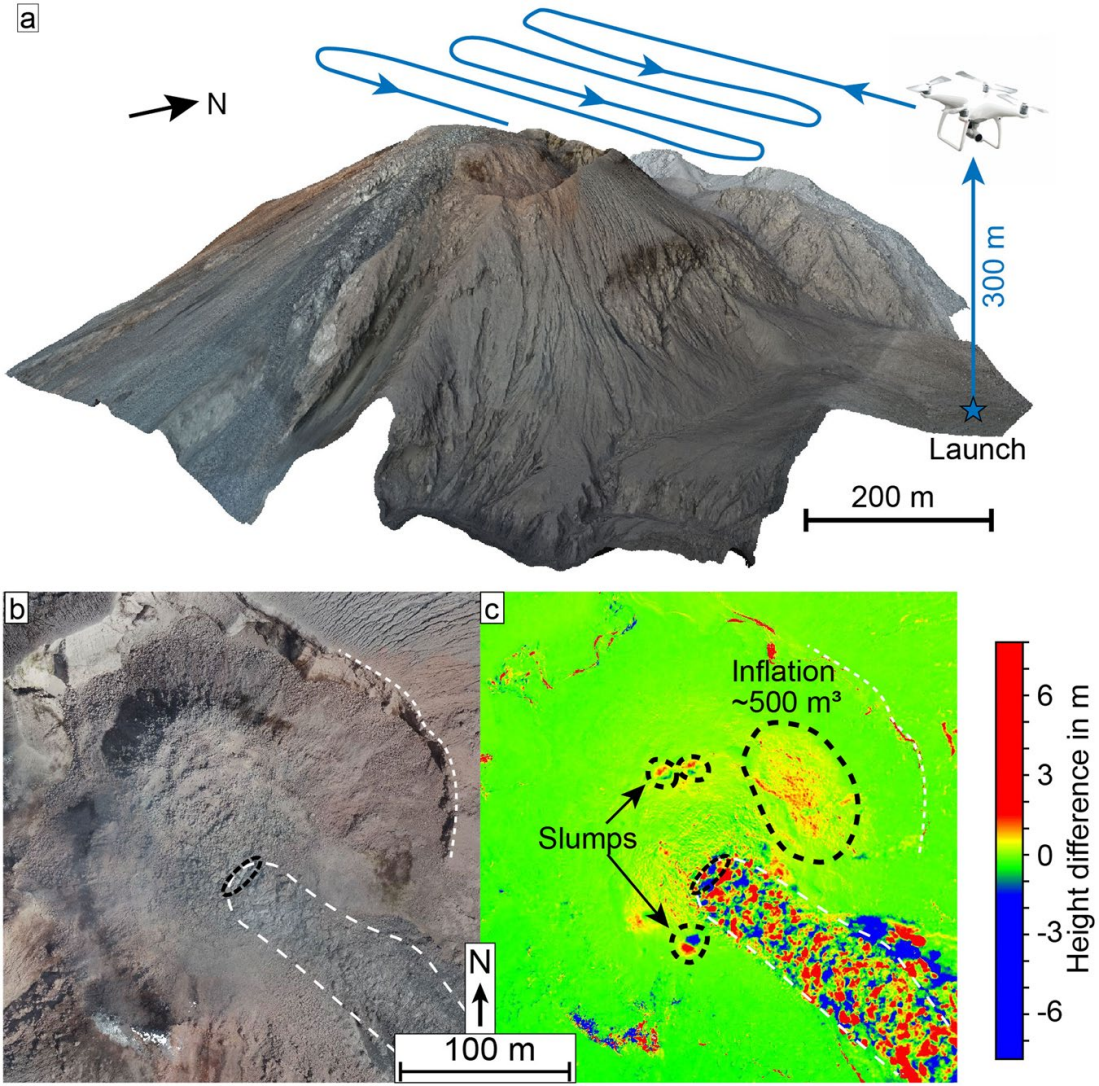
(**a**) A schematic of the UAS acquisition pattern and resulting SfM-model of a survey over the Santiaguito lava dome. The flight height was 300 m above the launch point and approx. 100 m over the top of the dome. (**b**) is an optical SfM-model of the dome surface and (**c**) a height difference map from two DEMs spanning a 3-day period (Survey C-D). The height difference map shows detailed surface changes, including an inflation as well as minor slumps on the dome surface. The scale is stretched to visualize the smaller deformation features against the large blocks moving with the lava flow. (a) was plotted in Agisoft Metashape (v1.5.2, www.agisoft.com) and (b,c) in CloudCompare (v.2.11, www.danielgm.net/cc/).

In this study, we remotely assess and characterize a lava dome and lava flow at Santa Maria volcano, Guatemala. A series of several adjacent lava domes are known as Santiaguito; the currently active one being called “Caliente” (Fig. 1a). The domes formed in the 1902 collapse crater of Santa Maria, one of the largest eruptions of the 20^th^ century^36^, with the remnant peak overlooking the domes. Santiaguito has displayed episodic lava dome growth since 1922^23^ and maintained eruptive activity until today, making it ideal to study lava dome processes. Previous studies revealed short term deformation processes associated with gas pressurization during eruptions^16^. Here, we used UASs equipped with optical and thermal infrared cameras (Fig. 2a) to image slow deformations of the lava dome surface. We construct DEMs and orthophotos, then trace the motions of pixels and use this data to delineate the 2D deformation field and gain insight on flow velocities, extrusion rates, surface strain, lava viscosity, and temperature anomalies over different time periods.

## Results

**Figure 3:**
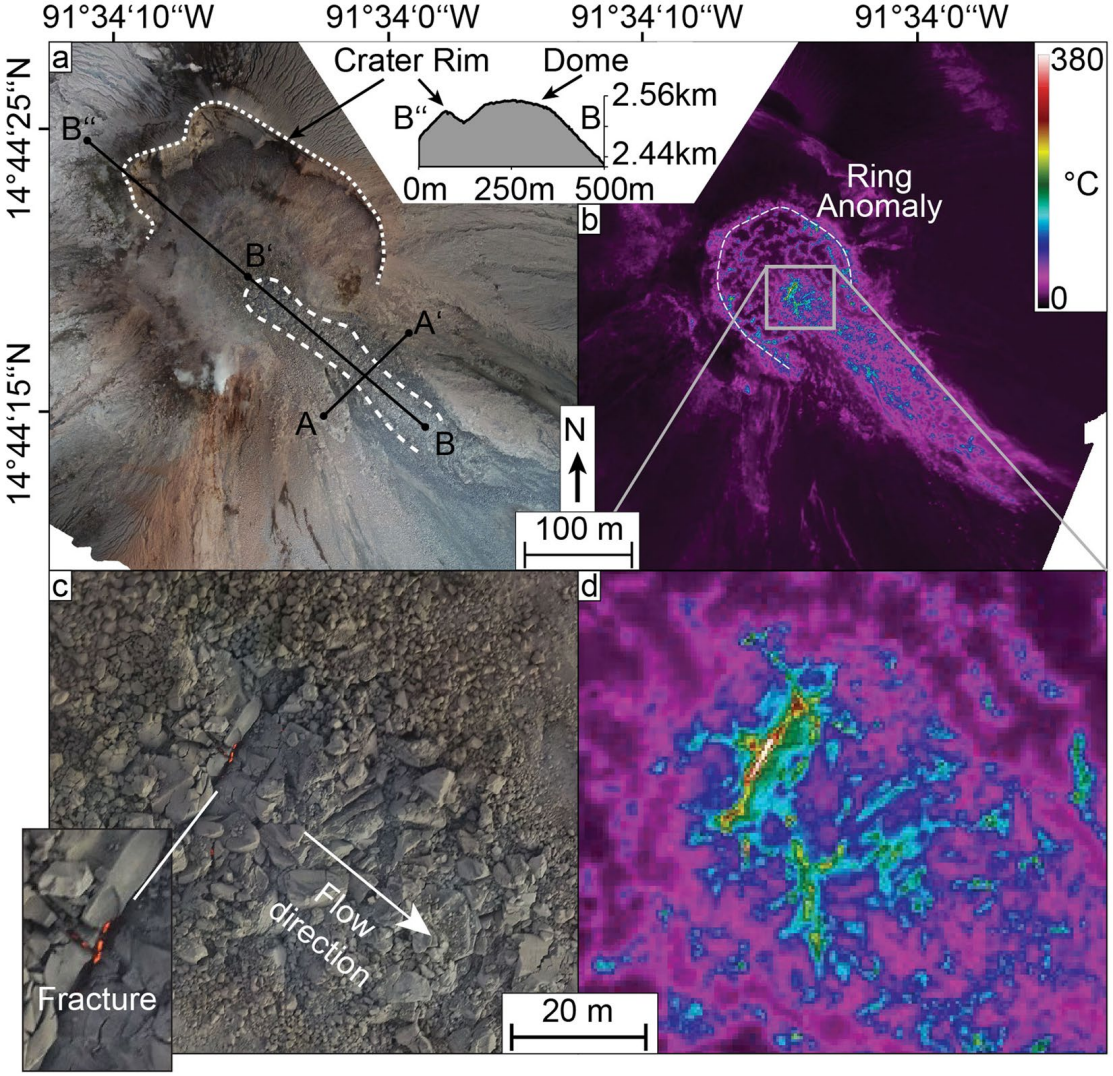
Optical (**a,c**) and thermal (**b,d**) orthophotos of the active Caliente lava dome and lava flow with a topographic profile. The profiles are further used in Fig. 6.

During fieldwork in February 2019 we performed repeated UAS surveys over the active “Caliente” lava dome, part of the Santiaguito complex at Santa Maria volcano, to assess dome morphology and structure. Topographic models generated with the SfM workflow show a flat-topped lava dome with steep sides and a blocky surface (Fig. 2a,b). The dome has a near-circular shape with a diameter of ~200 m and occupies an area of approximately 35,000 m^2^. It is situated within a larger explosion crater created in 2016^37^ and overtops this crater to the southeast, where a ~35 m wide lava flow is advancing down the flank (Figs. 1b, 2). The lava flow emerges from the summit dome as an embedded feature moving laterally. It then forms its own channel on top of the outer dome flank. The flow is also rather short as bits of the lower parts repeatedly broke away, resulting in minor rockfalls and very small pyroclastic flows on the north-east side. The thermal model, constructed from UAS-based thermal camera images, reveals high temperature areas on the dome, showing a ring-like temperature anomaly around the outer margins of the flat top (Fig. 3b), reminiscent of ring fractures previously observed at Santiaguito^38^. The lava flow is also distinguishable, having generally higher apparent temperatures compared to the dome surface (Fig. 3b). In the centre of the dome, a large fracture marks the start of the lava flow (Fig. 3c,d). This fracture is the thermally most active area and incandescence could be observed from it during darkness. It is also located orthogonal to the flow direction, suggesting it to be tension-induced from the lava flow movement.

Calculating height difference maps between the topographic models (spanning a 3-day period) reveals a distinct pattern of volume changes associated with the lava flow due to the movement of the large blocks on the surface (Fig. 2c), however, any overall volume gain associated with the lava extrusion could not be determined as the lateral lava extrusion did not cause major surface uplift and the lower parts repeatedly broke away. The flow thus effectively functions as a “conveyor belt” of lava from the dome down the flank and consequently the lava volume at the dome remains nearly constant. Moreover, we detect an inflation volume of ~500 m^3^ on the northeast side of the lava dome (Fig. 2c in the marked area) and a surface uplift of ~ 1 m. Due to the very low volume and the high signal to noise ratio from the lava flow block (some of which exceed 8 m in size) these values should be seen with caution. Some smaller slumps on the perimeter of the dome are also visible by the removal and addition of material in downslope direction, forming a radial pattern around the dome centre (Fig. 2c).

**Figure 4:**
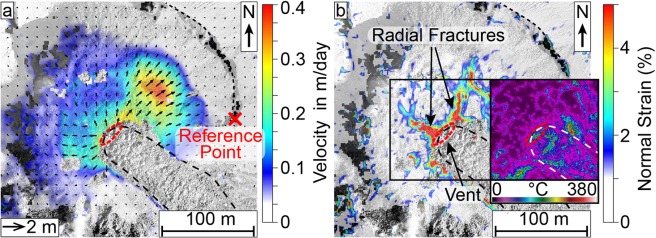
(**a**) deformation and (**b**) normal strain plot (normal in the image plane) for the lava dome expansion over the course of 3 days (Survey C-D), showing significant movement of the northeast dome side, as well as extending radial fractures near the lava flow vent. The arrow length in (a) shows the measured displacement. Survey C was performed on 15.02.2019 at 15:20-15:33 UTC and survey D on 18.02.2019 at 14:38-14:50 UTC. The lava flow and crater rim are marked for orientation. (b) also includes a thermal view on the vent, also captured on 18.02.2019 at 11:49-12:19 UTC. This shows that active structures of the lava flow produce significant thermal anomalies, however the radial fractures associated with the slower dome growth are not visible. The lava flow and crater rim are marked for orientation, the reference point for the shift and rotation correction is marked in (a). The figures were plotted in LaVision DaVis (v10.0.5.50575, www.lavision.de/de/products/davis-software/).

**Figure 5:**
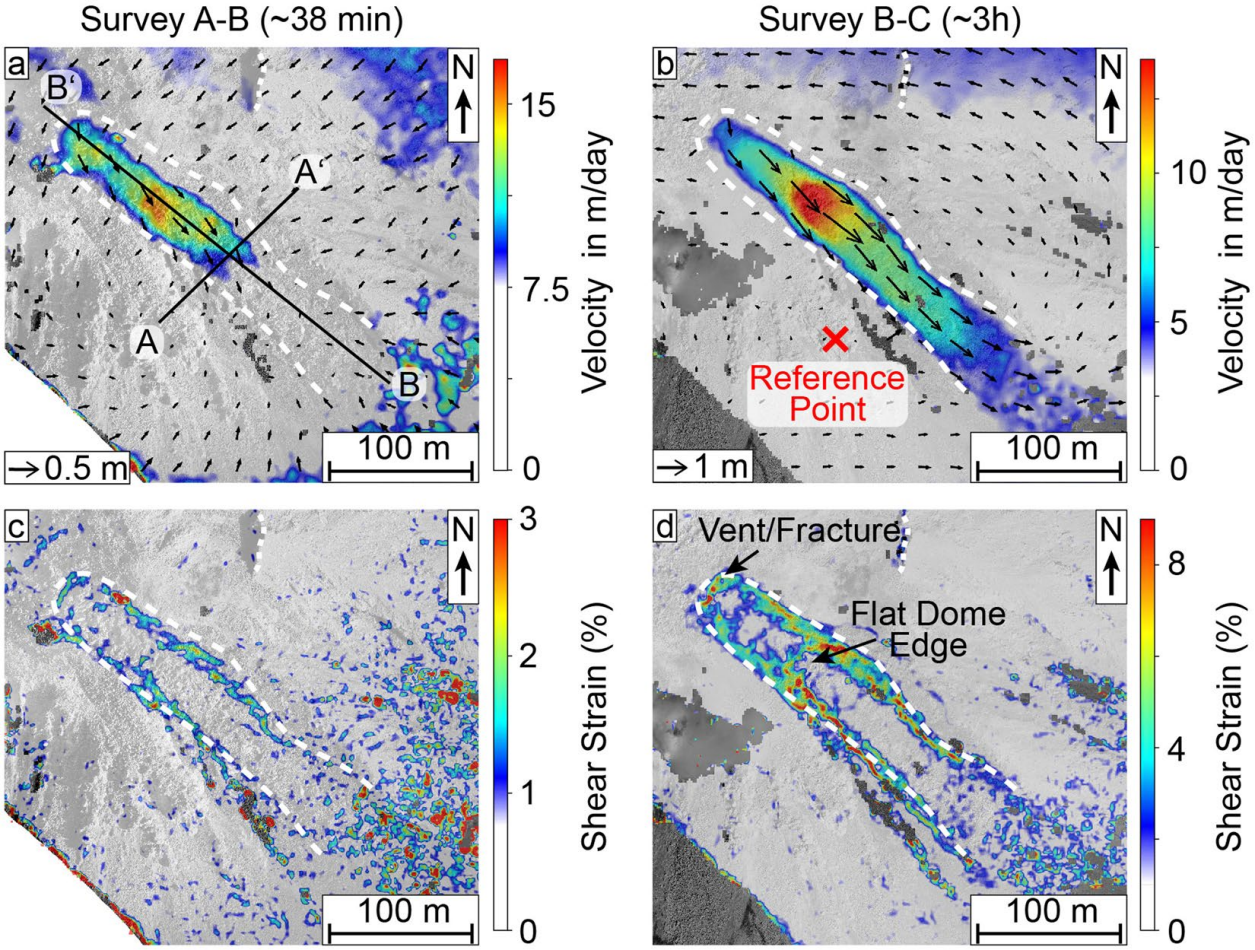
(**a**)-(**b**) flow velocity and (**c**)-(**d**) shear strain plots with 2D vector data for the lava flow plotted with DaVis. The arrow length in (a,b) shows the measured displacement. The flow is clearly distinguishable in all plots and the scales are adjusted to show the measured values over the background noise. The flights here were conducted on the 15.02.2019, survey A was performed at 11:52-12:18 UTC, survey B on 12:37-12:43 UTC and survey C at 15:20-15:33 UTC. The lava flow and crater rim are marked for orientation, the reference point for the shift and rotation correction is marked in (b). The figures were plotted in LaVision DaVis (v10.0.5.50575, www.lavision.de/de/products/davis-software/).

To gain further insight on the lava flow and dome movements and improve on the SfM analyses, we then tracked pixel-offsets in consecutive SfM-generated orthophotos using particle-image-velocimetry (PIV) (see methods) to track the motion of the lava dome surface, and measure surface deformation and flow velocities. This was applied on three pairs of consecutive orthomosaics with varying repeat times between flights (38 minutes, 3 hours and 3 days), all showing the dome from the same vertical perspective in high resolution. We note that small explosions occurred between surveys (Fig. 1b), however, no associated surface changes were observed and they did not noticeably affect our measurements. Results of the PIV show two separate scales of surface deformation; lateral expansion of the dome surface detectable over 3 days (Fig. 4a) and a lava flow motion detectable within minutes to hours (Fig. 5a,b). The lava flow could not be tracked over the flight interval of 3 days as the surface changes were too significant to allow feature based pixel tracking, resulting in decorrelation for that area (Fig. 4). Instead the tracking highlights a radial expansion of the dome surface moving from the centre outwards, but at much slower rates compared to the active lava flow (Figs. 4a, 5a,b). The magnitude of the expansion also coincides well with the volume increase detected by the DEM comparison (Fig. 2c). Localized expansion of the dome was largest on the north-east side with up to 1.2 m, equalling a velocity of 0.4 m/day (Fig. 4a). The expansion affects the dome only, whereas the crater appears stable. Moreover, the expansion establishes discrete zones of normal strain localization that emanate from the dome centre and the lava flow vent (Fig. 4b), which are otherwise not visible and do not show up as a thermal anomaly (Fig. 4b). The strain localizations are partitioning the dome carapace into 4-5 main blocks. The dome motion resembles a lateral spreading, but with a slight bulging on the north-east, represented by a maximum height increase of ~1 m (c.f. Figure 2c).

**Figure 6:**
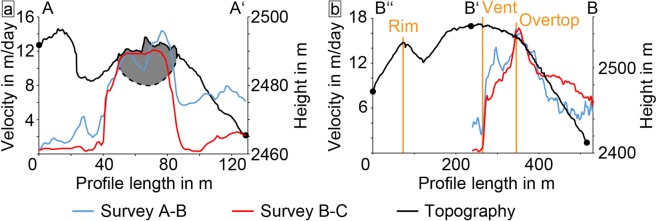
Profile for the measurements as marked in Figs. 3 and 5: (**a**) across the lava flow and (**b**) lengthwise over the dome and downhill on the flank. The estimated active flow field has been marked in grey. The flow depth is estimated from the flow thickness in the SfM data.

The shorter survey times of 38 minutes and 3 hours permit the tracking of the lava flow motion. While data is distorted towards the margins of the images (Fig. 5; see error discussion in methods), the lava flow is clearly distinguishable by its high displacement field. The flow vectors of the 38 min survey shows a maximum displacement of 0.4 m, corresponding to a velocity of approx. 15.2 m/day (Fig. 5a). For the 3 h survey (realized later on the same day), the maximum displacement is 2.0 m corresponding to a velocity of 15.4 m/day (Fig. 5b). The 3 h survey (Fig. 5b) generally shows the lava flow more clearly compared to the shorter 38 min survey, where movement is only detected in the upper flow portion (Fig. 5a). This is likely due to the higher absolute displacement over the longer survey time period (2.0 m and 0.4 m, respectively), causing a much more accurate and detailed result against the background errors. The active lava flow is further outlined with the computed shear strain, showing the margins of the flow as high strain regions and the flow centre as a low strain region (Fig. 5c,d). Moreover, high surface temperature regions correlate well with high shear strain regions at the flow margins as well as the flat dome edge (c.f. Figures 3b, 5c,d). A profile across the lava flow allows for a clear characterization of the flow dimensions, showing a sharp increase in deformation on the lava flow sides, but a stable value in the flow centre (Fig. 6a). The displacement velocity in flow direction shows a gradual increase beginning from the fracture and hitting a spike at the point of overtopping the flat dome surface onto the flank (Figs. 5a,b, 6b).

We gained further insight into the lava extrusion by selecting three representative points at different flow distances from the vent (Table 1, supplementary fig. S1) from the determined velocity fields (38 min and 3 h surveys) to derive the apparent viscosity of the lava flow. The apparent viscosity of the recent lava flow ranges from 2.6*10^9^ Pa·s to 1.3*10^10^ Pa·s and is very consistent between the two survey comparisons, with a notable viscosity increase with distance from the vent. Similarly, we extracted the extrusion rate from the channel dimensions and the lava flow velocity under the assumption that the measured flow rate is equal to the extrusion rate. The resulting values range between 0.04 to 0.06 m^3^/s.

**Table 1:** Summary of the viscosity η and flow/extrusion rate F calculation via the flow rate method. The bulk density ρ was inferred from Johnson *et al*.^16^, the height of the lava flow h, the flow half width a, and the slope α were measured using the SfM-Models. Since we could not measure the channel depth embedded in the flat dome top we assumed a similar 15 m based on the similar flow speeds and flow width compared to the downhill part. The data for individual Surveys can be found in the supplementary fig. S1, S3-8.

Variable	Survey A-B (Flat Top)	Survey B-C (Flat Top)	Survey A-B (Overtopping)	Survey B-C (Overtopping)
Time Difference	38 min	3 h	38 min	3 h
Displacement	0.33 m	1.24 m	0.43 m	1.99 m
ρ	2500 kg/m^3^	2500 kg/m^3^	2500 kg/m^3^	2500 kg/m^3^
g	9.81 m/s^2^	9.81 m/s^2^	9.81 m/s^2^	9.81 m/s^2^
h	~15 m (assumed)	~15 m (assumed)	~10 m	~10 m
a	~17 m	~17 m	~17 m	~17 m
α	~10°	~10°	~36°	~36° ± 3°
V	1.45*10^−4^ m/s	1.15*10^−4^ m/s	1.89*10^−4^ m/s	1.84*10^−4^ m/s
η	2.6*10^9^ Pa·s	3.3*10^9^ Pa·s	4.5*10^9^ Pa·s	4.6*10^9^ Pa·s
F	0.06 m^3^/s	0.05 m^3^/s	0.05 m^3^/s	0.05 m^3^/s
Variable	Survey A-B (Downhill)	Survey B-C (Downhill)		
Time Difference	38 min	3 h		
Displacement	0.32 m	1.15 m		
ρ	2500 kg/m^3^	2500 kg/m^3^		
g	9.81 m/s^2^	9.81 m/s^2^		
h	~15 m	~15 m		
a	~17 m	~17 m		
α	~39°	~39° °		
V	1.40*10^−4^ m/s	1.06*10^−4^ m/s		
η	9.7*10^9^ Pa·s	1.3*10^10^ Pa·s		
F	0.06 m^3^/s	0.04 m^3^/s		

## Discussion

The movement of the dome surface was observed in the form of two separate deformation processes with different timescales. Firstly, an expansion of the lava dome on the order of decimeters per day (Fig. 4), and secondly a lava flow with flow velocities on the order of several meters per day (Fig. 5). Both represent a different style of dome growth, the expansion and low volume increase indicate endogenous growth behaviour while the lava extrusion can be considered exogenous. Although we cannot rule out that these motions occur in sequence (e.g., first dome expansion, then lava extrusion or vice versa), it is more likely that they occur in association or even simultaneously. This is supported by the lava flow surface changing during the time the expansion was observed, and we did not observe any changes in the type of activity during our fieldwork (i.e., slow lava extrusion and low intensity explosions). Here, the expansion motion of the lava dome could be attributed to endogenous growth and lateral spreading supported by the volume increase on the northeast side (Fig. 2c), the radial outwards motion of the lava dome flanks (Fig. 4a), and the occurrence of radial fractures (Fig. 4b). Endogenous growth occurs by gradual intrusion of magma into the dome, which is non-reversible. This contrasts to deformation associated with short term gas-pressure changes described in previous studies^16^, which is reversible and represents a third timescale for potential dome deformation and was not observed here.

Interestingly, not all structures are associated with thermal anomalies. The vent fracture, the high shear strain regions and the edge of the flat dome top show high temperatures, likely due to the opening of fractures as a result of the lava movement (c.f. Figures 3b, 5). On the other hand, the radial fractures associated with the slower dome growth are not visible in the thermal infrared maps (Fig. 4b). This is either due to the slow opening of the fractures and effective cooling, or they do not provide pathways for hot fluids to escape.

Since the lava extrusion at Santiaguito occurred at nearly the same rates in both our measurements, our findings support the idea of very steady magma supply rates, which was hypothesized in earlier studies at this volcano^39^. The measured extrusion rates of 0.04-0.06 m^3^/s are very low, but consistent with previous assessments for Santiaguito in times of low activity^39^, although during more active cycles values have been observed to exceed 2 m^3^/s. Other studies also find generally higher extrusion rates, such as 0.5-1.6 m^3^/s between 2001 and 2002^1^, 0.2 in 2002^38^, and a mean of 0.43 m^3^/s between 2000 and 2009^40^. The extrusion rates at Santiaguito have thus slowed very significantly, although activity was still intermittently higher during 2012^40^.

**Figure 7:**
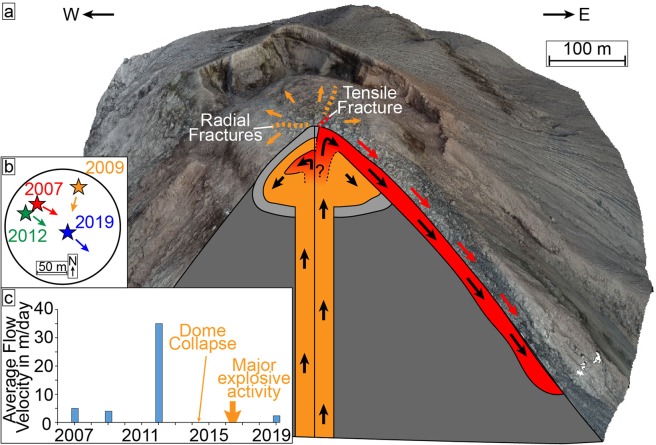
(**a**) Sketch of the inner lava dome structure inferred from our topographic and deformation data. The detected active lava flow extrusion and intrusion of the lava into the dome are marked in red, the current dome and conduit in orange. Fractures associated with both motions are also included. The grey area around the dome represents a brittle dome carapace. (**b**) Simplistic circle sketch representing the lava dome surface and showing the approximate vent position (star) and dominant flow direction for the different surveys. (**c**) Timeline plot showing the average dome surface flow velocities at four available data points in 2007, 2009, 2012 and 2019 (see supplementary fig. S2). A major dome collapse event and a phase of increased activity reported by Lamb, et al.^37^ are marked. (a) was plotted in Agisoft Metashape (v1.5.2, www.agisoft.com).

With the additional dome expansion, our measured extrusion rate may be slightly underestimating the magma influx into the dome, although judging by the very minor volume increase in the dome this is likely not significant. Part of the magma is extruded to feed the lava flow, whereas another part is intruded into the dome causing it to slowly expand (Fig. 7a).

Our calculations resulted in apparent viscosities on the orders of 10^9^ and 10^10^ Pa·s for the dome lava, which are similar to viscosity measurements determined for dacite lava flows at Santiaguito being on the same order of 10^9^ to 10^10^ Pa·s calculated by both the Jeffreys equation^1^ and measured in uniaxial compression experiments^20^. Studies on older lava flows (1930 and 1960s) further noted that lava viscosities increased with greater distance from the vent, but also measured lower lava viscosities of 5*10^7^ Pa·s in the 1930s^23^. In agreement with these studies, we also find a notable increase of the lava viscosity with greater distance from the vent, likely a result of the slow movement as well as gradual cooling and degassing, causing microlite crystallization and solidification, which can increase lava viscosity by up to several orders of magnitude^8^. Here, within less than 200 m flow distance, the viscosity of the 2019 lava flow increased by almost one order of magnitude. Thus, we determine that our data fit very well with previous assessments, and are in agreement with these studies within an order of magnitude.

Rheological investigations at other silicic dome building volcanoes indicate that viscosity may vary significantly. At Soufrière St. Vincent volcano, Lesser Antilles, theoretical estimations yielded a viscosity of 2×10^11^ Pa·s based on the rates of lateral spreading of the dome^41^. Rheological testing on several dome lavas from Volcán de Colima, México, Bezymianny, Kamchatka, and Unzen, Japan, showed values between approx. 10^9^ and 10^12^ Pa·s^42^. A lava flow originating from Volcán de Colima, México, in 1998-1999 measured 10^9^ to 10^10^ Pa·s^43^, which are closest to the rheology of Santiaguito. However, more well-established models^44^ predict viscosities of up to 10^14^ Pa·s, but are difficult to compare to dome lavas as the viscosities will have a broad range strongly depending on the state of cooling, outgassing, and crystallization. These factors also influence the flow regime, with our viscosity calculations assuming the lava to behave as a Newtonian fluid^45^. Since the occurrence of high shear stress is limited to the margins of the flow (Fig. 5c,d), the latter assumption is likely valid for the inner parts of the flow, where we made the measurements, with the shearing at the channel margins acting as a conveyor belt. However, a transition to non-Newtonian behaviour must occur on the lower flank since the repeated breaking-off of the lava flow front indicates brittle behaviour under the acting strain rates.

The seemingly rather low apparent viscosity (compared to other silicic lavas) observed at Santiaguito in 2019 generally agrees with the ongoing dome extrusion characteristics with only low intensity explosions. However, changes in lava viscosity resulting from changing extrusion rates, increased cooling or degassing can drastically reduce the free escape of volatiles, resulting in increased explosive activity^8^. Particularly, changes in viscosity will affect the failure mode (brittle-ductile) of the dome magma and control surface strain and, in turn, the development of fractures on the dome surface^46^. Changing viscosity may also impact the mode of extrusion between endogenous and exogenous growth^47^.

We compared our UAS results to previous ground-based imaging realized from the top of the adjacent Santa Maria summit (see supplementary fig. S2). This position provides an oblique view on the top of the Caliente dome, and allows for an estimation of the flow velocities on the dome surface. From this distance, we could not determine the growth of the dome, but we could measure the ongoing lava extrusion. Here, data is available from 2007, 2009, and 2012 showing that the lava flow had velocities on the order of 2-5 meters per day (Fig. 7c). A more active period was observed in 2012 with flow speeds being on the order of 30-40 meters per day (Fig. 7c), which is in agreement with observations from other campaigns in that period^40,48^. While no flow velocity data is available after 2012, other studies report low activity, aside from a major dome collapse in 2014^37^. The low activity lasted until early 2016, which was characterized by both strong explosions and increased lava extrusion^37^. This high activity ceased in late 2016 and by 2019 activity had reduced to similar flow velocities as before 2012 (Fig. 7c). Such transitional changes are a common trend at Santiaguito since long periods of low and mostly effusive activity are intermitted by periods of heightened explosive activity^37,39,48^. An extrusion rate could not be calculated for the ground-based imaging as no detailed topography data are available to characterize the flow channels. Here, our data only covers short observation intervals, so the lava flow velocities or extrusion rate may vary significantly, but the consistent measurements during low activity further suggest the lava flow to be fed from a rather constant magma supply. More regular survey data could help to identify, characterize, and potentially forecast such changes in activity.

We further observe that the lava flow directions have varied only slightly and are dominantly towards the southeast, only being directed south in 2009 (Fig. 7b). This is surprising since similar instances of increased explosive activity at other dome-growing volcanoes such as Volcán de Colima, Mexico, can reshape the edifice morphology and change lava flow directions^19^. The persistent morphology and activity at Santiaguito suggest that the shallow structures affecting dome growth and flow direction are also persistent and largely unaffected by more intense eruptive phases. This is likely a result of the relatively low lava viscosity compared to other dacite lavas as well as the open outgassing conditions attributed to Santiaguito^37,49,50^. Furthermore, it is likely that the magma plumbing system at the Caliente dome has not undergone repeated changes to magma-pathways as comparable dome-building volcanoes such as Volcán de Colima^19,51,52^, although the entire Santiaguito dome complex has formed due to such changes over the course of the last 100 years. However, changes in the degassing system, e.g., the permeability by strain localisation in the conduit^53^, will affect the explosivity of the volcano and should be monitored closely to predict potential changes.

Explosive activity (Fig. 1b) can considerably reshape the surface of a lava dome, or crater, and may introduce potential artifacts into our analysis. We counted the number of explosions during the study period using the cyclic inflation signals from a tiltmeter station deployed near the dome and our UAS-launch site. One explosion occurred during the 38 min survey, 2 during the 3 h survey, and 22 during the 3 days survey. By visual observations we find that the explosions were of low intensity without any ballistics. Here, the explosions occurring during lava extrusion are thus more reminiscent of degassing pulses through a relatively open system and it is unlikely that they significantly impacted the surface morphology of the dome and we observed no changes in our survey data. Hence, we do not think that the explosive activity affected our results.

As this study demonstrates, displacements of the lava dome surface occur on different scales. Campaigns using fixed cameras at the summit of Santa Maria in the past years have documented both very rapid dome uplift (10^0^-10^1^ s) coinciding with explosions^16^ as well as slower processes (10^2^ to 10^3^ s) associated with localized bulging of the dome surface. For example, in 2012 a ten-minute transient bulging event with uplift of several meters was proposed using a 1-minute time lapse sequence from a fixed summit camera^50^. While the repeat survey time and relatively low activity of the Santiaguito lava dome during this study did not allow us to capture such movements, we could discriminate variations in surface motion of extruding lava as well as radial expansion and bulging of the dome by intrusive growth. Considering the potentially severe hazards displayed by growing lava domes, we also note that UASs represent a fast and safe way to gather important data such as dome deformation and lava properties as demonstrated in this study. This makes them an excellent tool to assess the activity and hazard potential of an active lava dome, especially in crisis situations.

## Methods

### Fieldwork

During our fieldwork in February 2019 we performed UAS survey flights over the active Caliente vent at Santiaguito volcano with multiple drones and sensors at a height of approx. 100 m over the lava dome (Figs. 1a, 2a). The flights were recorded using high-resolution optical photos and an additional thermal camera. The optical images were acquired with the onboard sRGB camera of our UAS (DJI Phantom 4 Pro) with a resolution of 5472 ×3078 px and a sampling rate of 0.5 Hz. The thermal images were acquired with a FLIR TAU 2 (9 mm lens), stored in a TEAX ThermalCapture frame grabber, and processed with the Thermoviewer software (v3.0.4). These provided radiometric temperature data and had a resolution of 640 ×512 px with a sampling rate of 8 Hz. Four flights could successfully be processed into 3D-Models and yielded good results via PIV (named surveys A, B, C and D). These were recorded over different timespans of 38 min (A-B), 180 min (B-C) and ~3 days (C-D).

### Tri-stereo photogrammetry using Pléiades satellite data

To georeference the UAS datasets acquired at Santiaguito volcano, we tasked high-resolution optical Pléiades satellite imagery on 15.01.2017. Its tri-stereo capability with one near-nadir and two off-nadir images enables the construction of a highly detailed topography model of the volcano and its surrounding. We processed the three panchromatic images with the ERDAS IMAGINE Photogrammetry toolbox similar to the working process described by Bagnardi, *et al*.^54^, post-processed the resulting point cloud with CloudCompare, and gained a topographic model with a spatial resolution of 5 m/px.

### SfM-Photogrammetry

We performed Structure-from-Motion (SfM) photogrammetric processing^55^ using Agisoft Metashape 1.5.2 on all optical and thermal surveys to reconstruct the 3D environment of the active lava dome (Fig. 2a), enabling us to construct both high resolution DEMs from dense point clouds as well as detailed orthophotos. The survey photos were acquired in nadir or near-nadir position, with only some single photos being taken at an oblique angle to improve the model quality^56^. The difficult and hazardous terrain did not allow for sufficient ground control points to create a reliable georeferencing. Instead, we utilised the photogrammetric DEM built from Pléiades data for more accurate and consistent georeferencing by point matching the models. In this process, the SfM-built DEM is attached to the Pléiades one by identifying recognizable features in both datasets. The final referencing had an RMS-error of 3.3 m, but due to the high resolution of the SfM models, they could be better referenced relative to each other, meaning that all other SfM-DEMs now use the prereferenced Survey D as a basis. This minimizes the relative errors between the DEMs and allows for high-precision data comparison. Here, all models are within 0.3 m accurate relative to each other (see supplementary fig. S3-8).

In order to allow feature tracking, orthophotos (georeferenced nadir photos) of the study area are generated by projecting the survey photos onto the 3D-surface of the DEMs. While this reduces 3D data to 2D, it has the advantage that the image texture resolution of the resulting photo is increased and thus exceeds the spatial resolution of the DEMs, allowing to resolve more detailed ground features and motions in a single large photo. The orthophotos can be found in the supplementary fig. S3-8 and the DEMs can be seen as hillshade maps in supplementary fig. S9-11.

### Particle image velocimetry

For the pixel-offset tracking we employed a particle-image-velocimetry (PIV)^57^ method for the four high-resolution orthophotos taken from the SfM workflow using LaVision DaVis 10.0.5 to track and calculate changes on the lava dome and lava flow surface based on the intensity value of the orthophotos. This enabled us to accurately quantify the velocity and direction of deformation and flow on the active dome. The maximum normal and shear strain values were also calculated based on the eigenvalues of the 2D stress tensor (at 45° for the shear strain), allowing insight into the nature of deformation. Similar techniques have previously been applied to characterize landslide^58^ and glacier^59^ motions using either UAS-based photos or satellite images. Here, we add to this method by measuring very low displacement motions in high detail and over different timescales.

### Apparent viscosity calculation

Combined with the 3D terrain reconstruction from the SfM workflow we are further able to constrain all parameters required to estimate the lava viscosity based on the Jeffreys equation^60^. This has been used to characterize lava viscosity in several previous studies^1,22,61,62^. Here we used the formula applied to the surface flow behind the flow front adapted to a semi elliptical channel shape after Moore^61^, which is best suited to account for the unknown channel geometry^63^:

1 $$\eta =\rho \cdot g\cdot \,\sin (\alpha )/({\rm{V}})\cdot \{\frac{{{\rm{h}}}^{2}}{2\cdot [{({\rm{h}}/{\rm{a}})}^{2}+1]}\}$$

where η is the apparent viscosity of the lava, *ρ* the bulk density, *g* the gravity acceleration, *h* the thickness of the lava flow, *a* the flow half-width, *α* the slope of the surface, and V the velocity of the flow. The flow rate F was further calculated as a representative value for the extrusion rate, assuming the two values are equal, by multiplying the semi-elliptical channel cross-section area with the measured flow speed.

2 $${\rm{F}}=\frac{{\rm{\pi }}\cdot {\rm{h}}\cdot {\rm{a}}}{2\cdot {\rm{V}}}$$

This was applied to the three points of the lava flow representative of the flow movements, one near the vent on the flat top of the dome, one near the overtopping of the breached crater to the southeast, and one down the flank. We used the points where the deformation was largest to avoid influences by channel shear and represent the lava flow parts most unaffected by cooling and solidification.

### Errors and data accuracy

The UAS orthophotos achieved a resolution better than 7 cm, allowing the recognition of single blocks and fractures. The thermal data provided lower resolutions of 41 cm or better, but allowed additional insight into the apparent surface temperatures. The measured apparent temperatures can vary depending on a number of factors^64^ including object emissivity, humidity, surface roughness, acquisition angle, object distance, solar radiation, and the presence of ash or gas in the atmosphere. Here we assume similar conditions to previous studies at lava domes and correct the atmospheric attenuation in the Thermoviewer software based on an emissivity of 0.95, a transmissivity of 0.7 as well as environmental and path temperatures of 10 C°^65,66^. While the temperature measurements are as accurate as possible, the values should still be seen with caution, as some factors could not be considered for correction. One factor impacting our results is the acquisition angle, the sensor is mounted vertically to the UAS but tilts slightly with the drone during flight. This causes drifts in the measured temperatures on the order of a few degrees. It is also likely that some temperatures are underestimated due to the sub-pixel imaging of features like the tensile fracture on the dome top (Fig. 3c,d). Here we kept the apparent temperature measurements as accurate as possible by conducting the thermal surveys before sunrise, thus omitting artifacts from solar heating or reflection.

As a result of the georeferencing errors, a slight shift between compared survey orthophotos is expected. Similar SfM-based studies^19,34,58^ commonly achieve accuracies on the order of cm to dm and ours have an error of less than 0.30 m for the optical models. However, the maximum lava flow movement between Survey A-B was just above that with about 0.43 m. To maximize the distinction between moving and stationary components, we employed an additional shift and rotation correction based on contrast-rich stationary points (here, the crater rim or the volcano flank, see Figs. 4, 5), thus further optimizing the accuracy of the PIV. As a result, the deformation values are very reliable, and an estimate for the systematic shift error can be taken from the background values in the lava flow surrounding (approximately of the order of 0.01-0.10 m). Despite the additional shift and rotation correction, we still note an increasing error towards the margins of our models (Fig. 5a,b). We attribute this to limitations in the SfM reconstructions on the borders of a photogrammetric model, where a lower degree of overlap between photos causes a lower accuracy of the reconstructed points. In the case of the shorter survey with 38 minutes the resulting lower effective displacements make the survey more susceptible to such background errors. However, since we picked our velocity data mostly in the central areas of the models, our measurements should still be reliable and our measurements are likely not affected to a large degree as is confirmed by the very similar measured flow speeds.

The largest expected errors for the Jeffreys equation are systematic in nature (i.e. the margin effects) and we picked our values either from the SfM-models or the PIV data. We tested the robustness of our calculations by systematically varying the input parameters. We find that with the input parameters being accurate to within ~20% of their value, our calculations are accurate to within an order of magnitude. This makes our approach an excellent tool to gain quick first order data on lava viscosity and it should be suitable to assess the type of activity and potential hazards at erupting volcanoes.

## Supplementary information

Supplementary Information.

Supplementary Figure S9.

Supplementary Figure S10.

Supplementary Figure S11.

## Data Availability

The datasets generated during and/or analysed during the current study are available in the GFZ Data Services^67^ under 10.5880/GFZ.2.1.2020.001. This includes the UAS orthophotos, DEMs and point louds. Alternatively, the datasets are available from the corresponding author on request.
