# Supplementary material for: UAS-based tracking of the Santiaguito Lava Dome, Guatemala
Source: Sci Rep. 2020 May 25;10:8644. doi: 10.1038/s41598-020-65386-2 (PMC7248112; doi:10.1038/s41598-020-65386-2)

Supplementary Material:

UAS-based tracking of the Santiaguito Lava Dome, Guatemala

Edgar U. Zorn*[1], Thomas R. Walter[1], Jeffrey B. Johnson[2], René Mania[1]

[1]German Research Centre for Geosciences GFZ, Telegrafenberg 14473 Potsdam, Germany

[2]Boise State University, Department of Geosciences, Boise, ID, United States

Figure S1: The Figure shows (a) the measurement points (red dots) for the apparent viscosity calculations and profiles across the flow channel at these points. Particle divergence plotting (b) also shows the relative lava flow motion, assisting in picking appropriate points representative of different flow regions. It shows stretching near the large fracture at the dome centre and on the edge of the flat top near the overtopping onto flank, where the flow bends downwards as it transitions to the flank, minor compression follows below. The corresponding profiles are shown in (c-e). (b) was plotted with LaVision DaVis (v8.4.0, www.lavision.de/de/products/davis-software/).

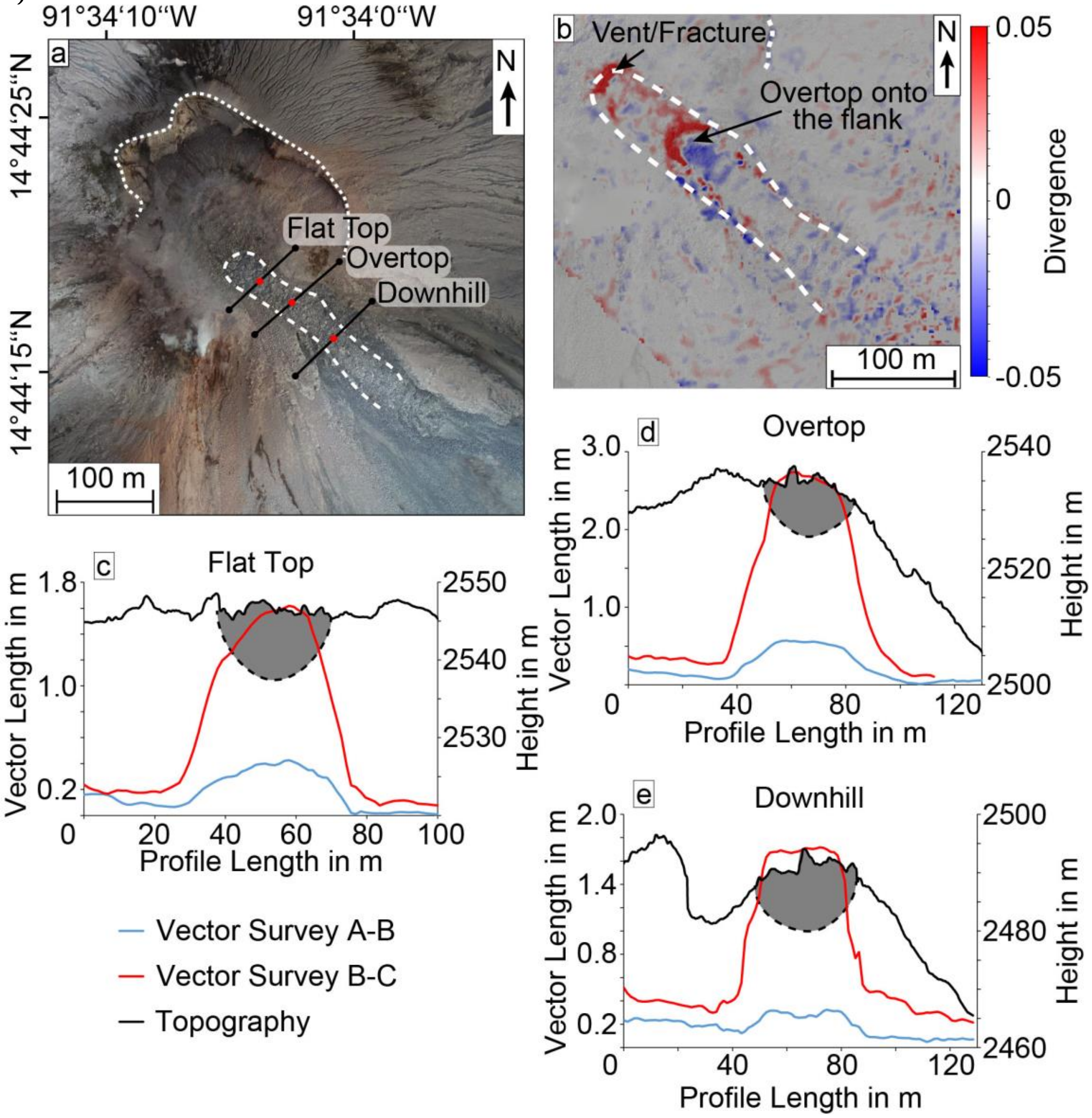

Figure S2:

Time lapse PIV in (a) 2007, (c) 2009, and (e) 2012 recorded from the Santa Maria summit was used to measure surface movements on the flat top of the lava dome over 4-hour time spans. A look angle of 30 degrees (relative to horizontal) was assumed and used to estimate vector amplitudes projected on to a horizontal plane. Lava flow motions were calculated from 2-D image cross correlation in the spatial frequency domain. Vector fields were then interpolated and smoothed. Yellow dash-dot line annotations indicate approximate boundaries of active and stagnant lava. Solid yellow lines indicate fracture zones and surface lava rifting. Yellow stars correspond to approximate locations of vertical upwelling. Histograms (b), (d), (f) are shown for vector fields within the yellow dash-dot lines and are scaled to 1-day (24-hour) motions. The same histogram (h) is calculated based on the current vectors measured by UAV in this study (g). The area was masked to only include the flat dome top for this purpose to keep the data comparable and to avoid unmoving areas in the measurements.

The data shows that the type of activity at the Caliente lava dome has stayed very consistent throughout the last decade as the dome and lava flow show the same features as in 2019, these being the flat-topped dome inside the main crater and a constant lava flow down one side. The velocity of the lava flows since 2007 is on the order of several meters per day (b,d,f), which is very consistent with our new data from 2019 (g-h). A more active period is observed in 2012 with flow speeds being on the order of tens of meters per day (e-f). Another notable observation is the distribution of flow movement. In 2007 and 2009 large portion of the dome surface moved evenly and resulted in a rather uniform distribution of velocity vectors (b,d). This changed in 2012, where high velocity areas became more localized and resulted in few high velocity vectors and many low velocity ones as the flow area narrowed (e,f). In 2019 this is even more apparent as the lava flow moves in a single narrow channel, with the rest of the dome surface only moving slowly instead (g,h). We thus conclude that the UAV-based data is suitable to be compared with and improve upon previously captured image data.

(a,c,e) were plotted in MATLAB R2018b (v9.5.0, de.mathworks.com/products/matlab.html) and (g) was plotted in LaVision DaVis (v10.0.5.50575, www.lavision.de/de/products/davis-software/).

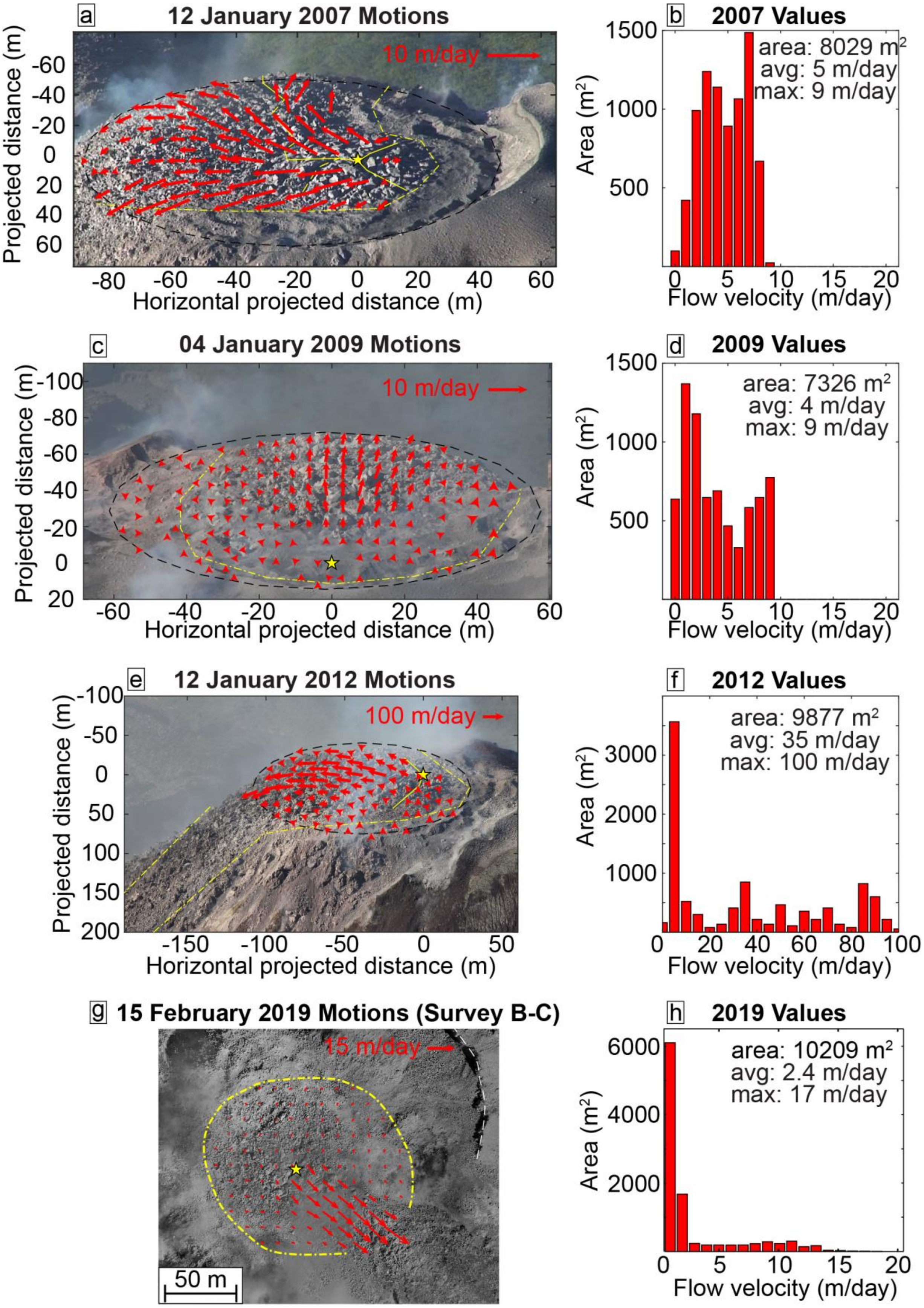

a
12 January 2007 Motions
10 m/day
Projected distance (m)
Horizontal projected distance (m)
b
2007 Values
area: 8029 m²
avg: 5 m/day
max: 9 m/day
Area (m²)
Flow velocity (m/day)
c
04 January 2009 Motions
10 m/day
Projected distance (m)
Horizontal projected distance (m)
d
2009 Values
area: 7326 m²
avg: 4 m/day
max: 9 m/day
Area (m²)
Flow velocity (m/day)
e
12 January 2012 Motions
100 m/day
Projected distance (m)
Horizontal projected distance (m)
f
2012 Values
area: 9877 m²
avg: 35 m/day
max: 100 m/day
Area (m²)
Flow velocity (m/day)
g
15 February 2019 Motions (Survey B-C)
15 m/day
50 m
h
2019 Values
area: 10209 m²
avg: 2.4 m/day
max: 17 m/day
Area (m²)
Flow velocity (m/day)

The survey orthophotos can be found on the following pages. The provided georeferencing RMS represents the relative referencing errors between the point clouds (relative to Survey D). All clouds have an additional error of 2.93 m to their absolute geolocation indicated by the point matching to the Pléiades satellite data (see methods).
The data was generated with Agisoft Metashape (v1.5.2, www.agisoft.com).

Supplementary Figure S3 (Survey A):
15.02.2019 at 11:52-12:18 UTC with DJI Phantom 4 Pro
SfM spatial Resolution: 13.3 cm
Orthophoto texture resolution: 18330 by 18816 px, 6.65 cm/px
Relative georeferencing RMS: 0.28 m

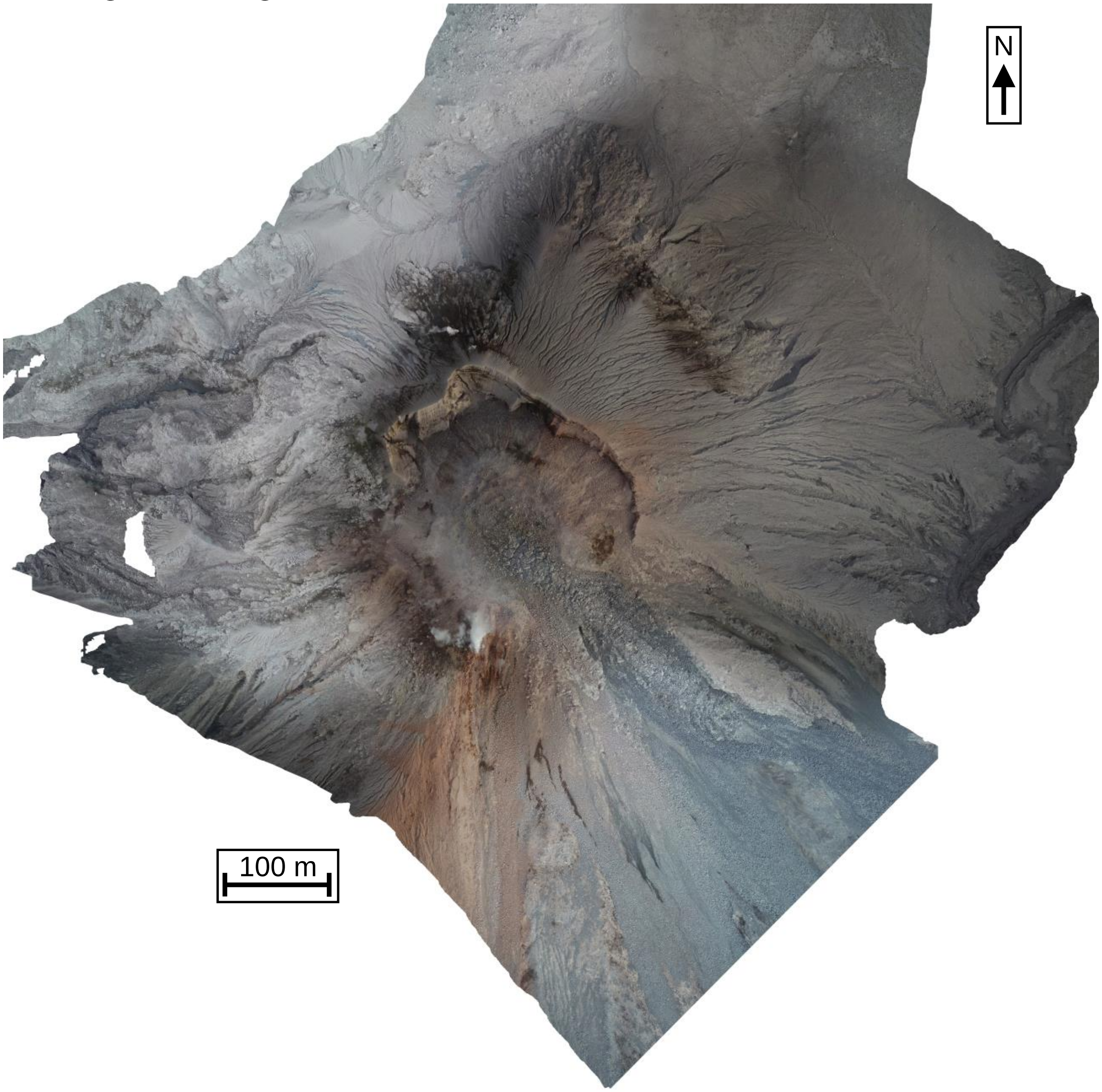

Supplementary Figure S4:
15.02.2019 at 12:12-12:25 UTC with FLIR TAU 2
SfM spatial Resolution: 40.9 cm
Orthophoto texture resolution: 2977 by 3056 px, 40.9 cm/px
Relative georeferencing RMS: 0.27 m

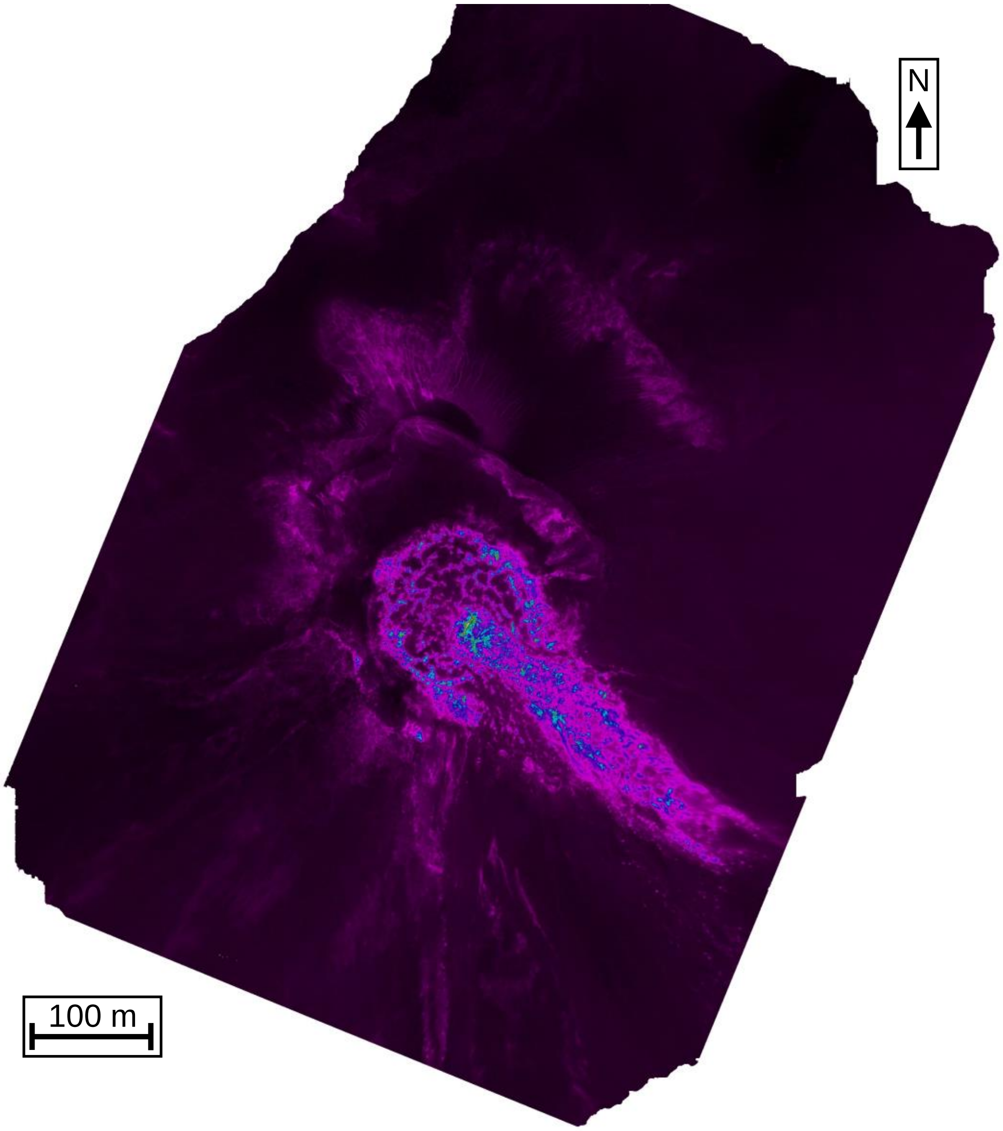

Supplementary Figure S5 (Survey B):
15.02.2019 at 12:37-12:43 UTC with DJI Phantom 4 Pro
SfM spatial Resolution: 10.6 cm
Orthophoto texture resolution: 22925 by 23534 px, 5.32 cm/px
Relative georeferencing RMS: 0.27 m

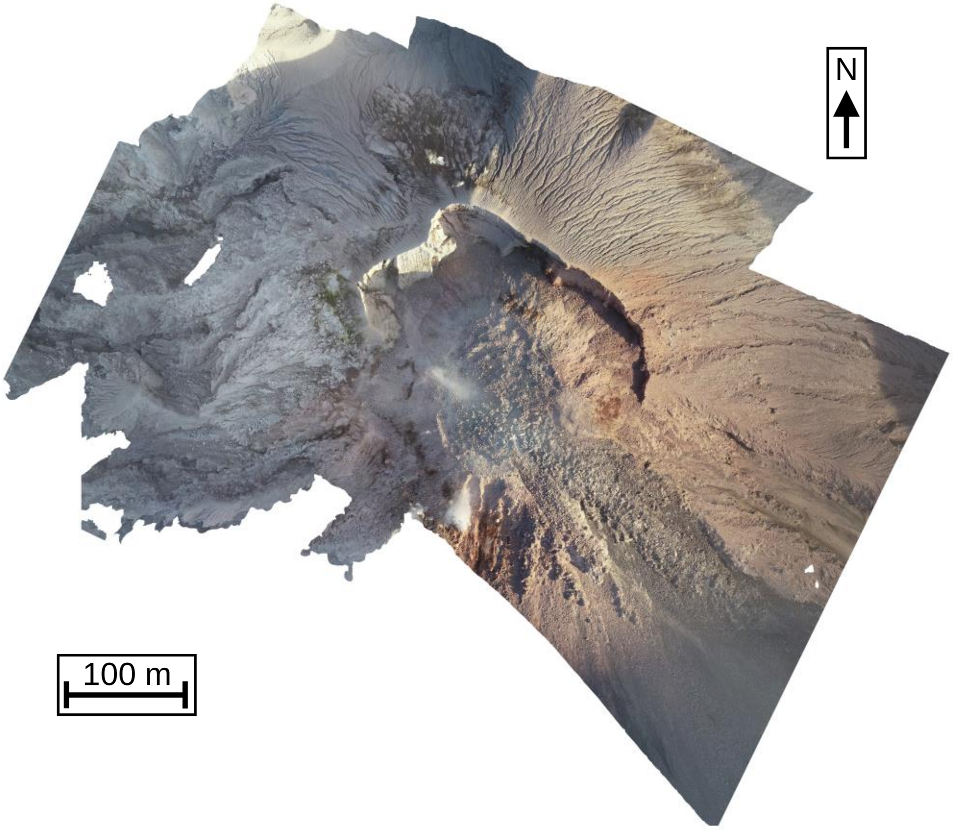

Supplementary Figure S6 (Survey C):
15.02.2019 at 15:20-15:33 UTC with DJI Phantom 4 Pro
SfM spatial Resolution: 13.3 cm
Orthophoto texture resolution: 18325 by 18811 px, 6.65 cm/px
Relative georeferencing RMS: 0.27 m

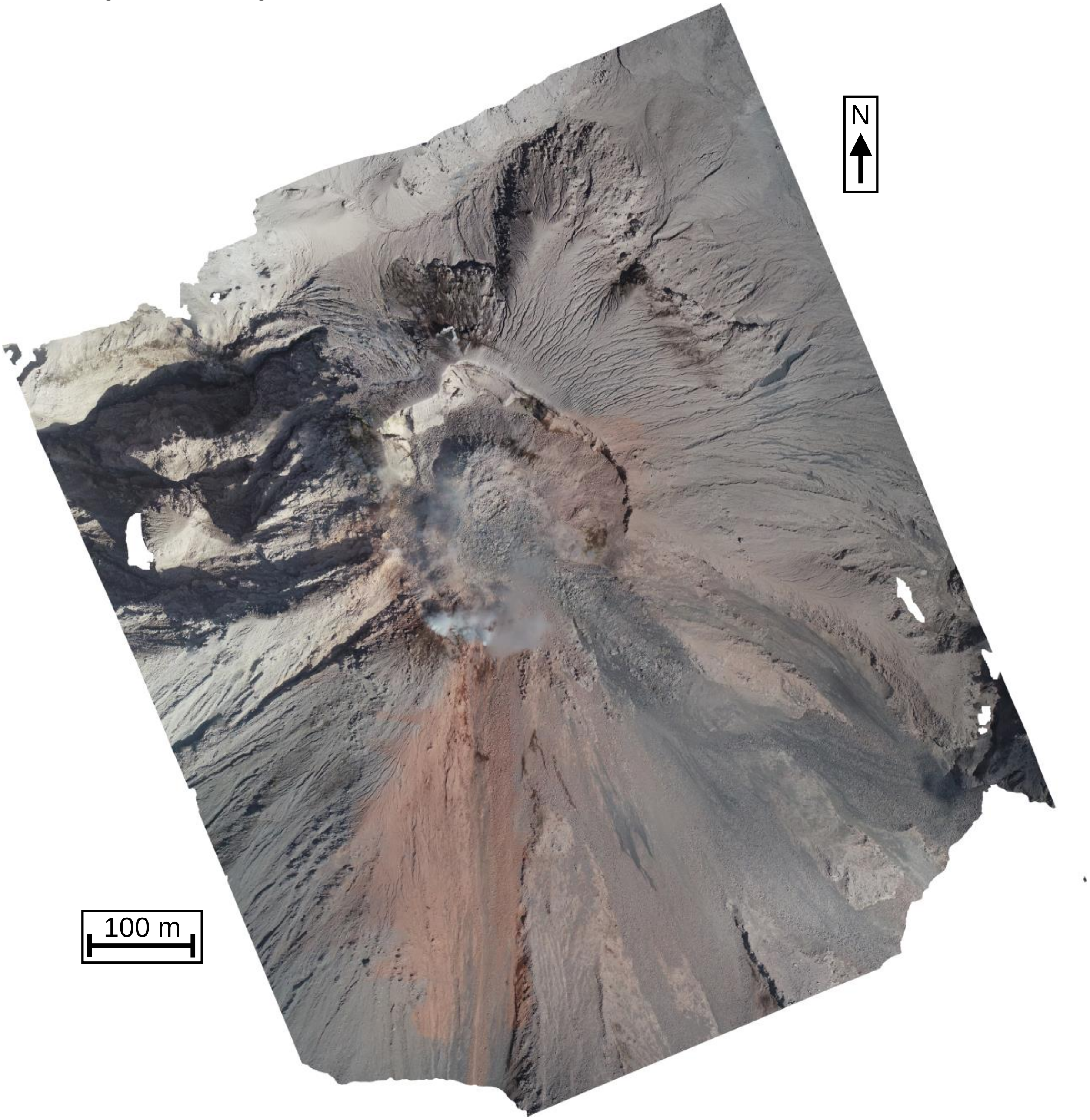

Supplementary Figure S7:
18.02.2019 at 11:49-12:19 UTC with FLIR TAU 2
SfM spatial Resolution: 27.1 cm
Orthophoto texture resolution: 4496 by 4615 px, 27.1 cm/px
Relative georeferencing RMS: 0.16 m

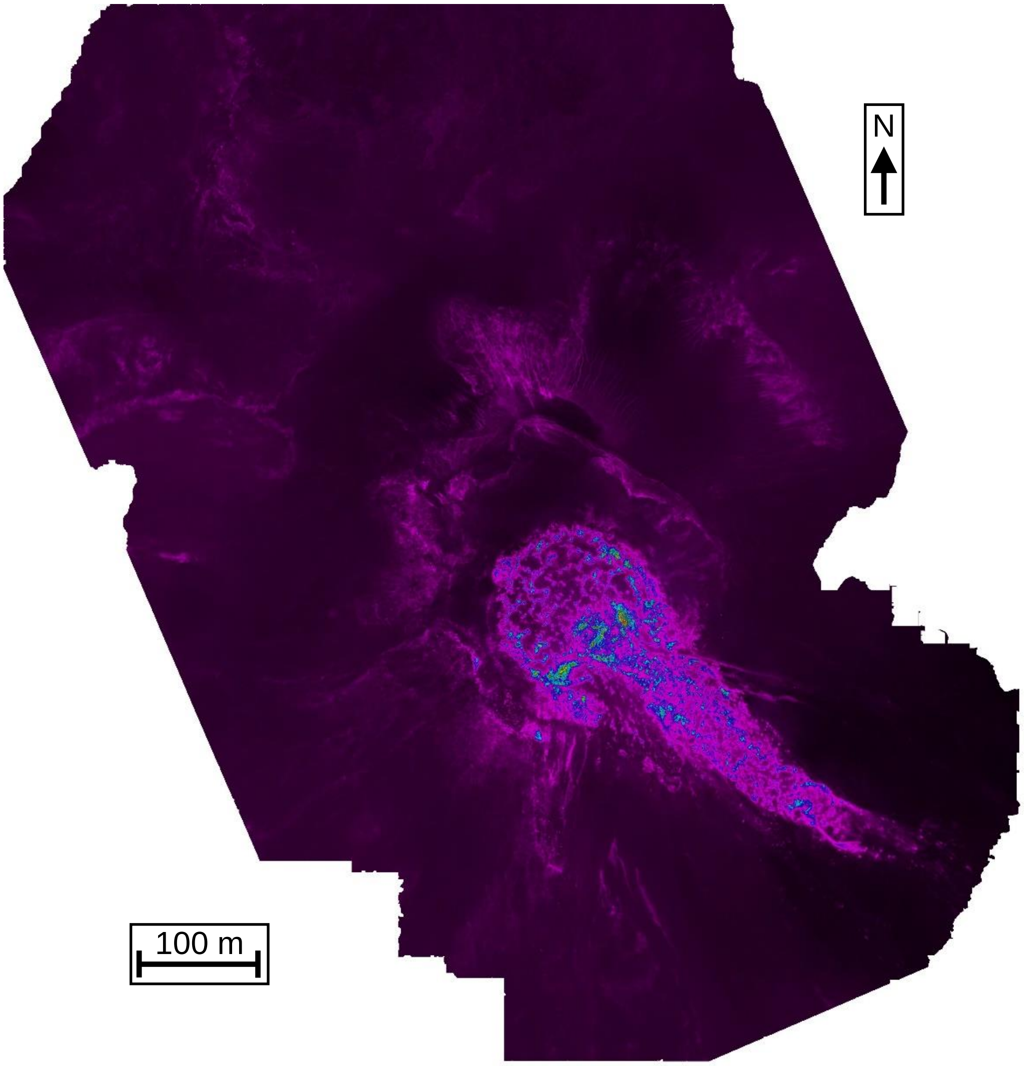

Supplementary Figure S8 (Survey D):
18.02.2019 at 14:38-14:50 UTC with DJI Phantom 4 Pro
SfM spatial Resolution: 11.3 cm
Orthophoto texture resolution: 19651 by 20173 px, 6.20 cm/px
Relative georeferencing RMS: Not applicable

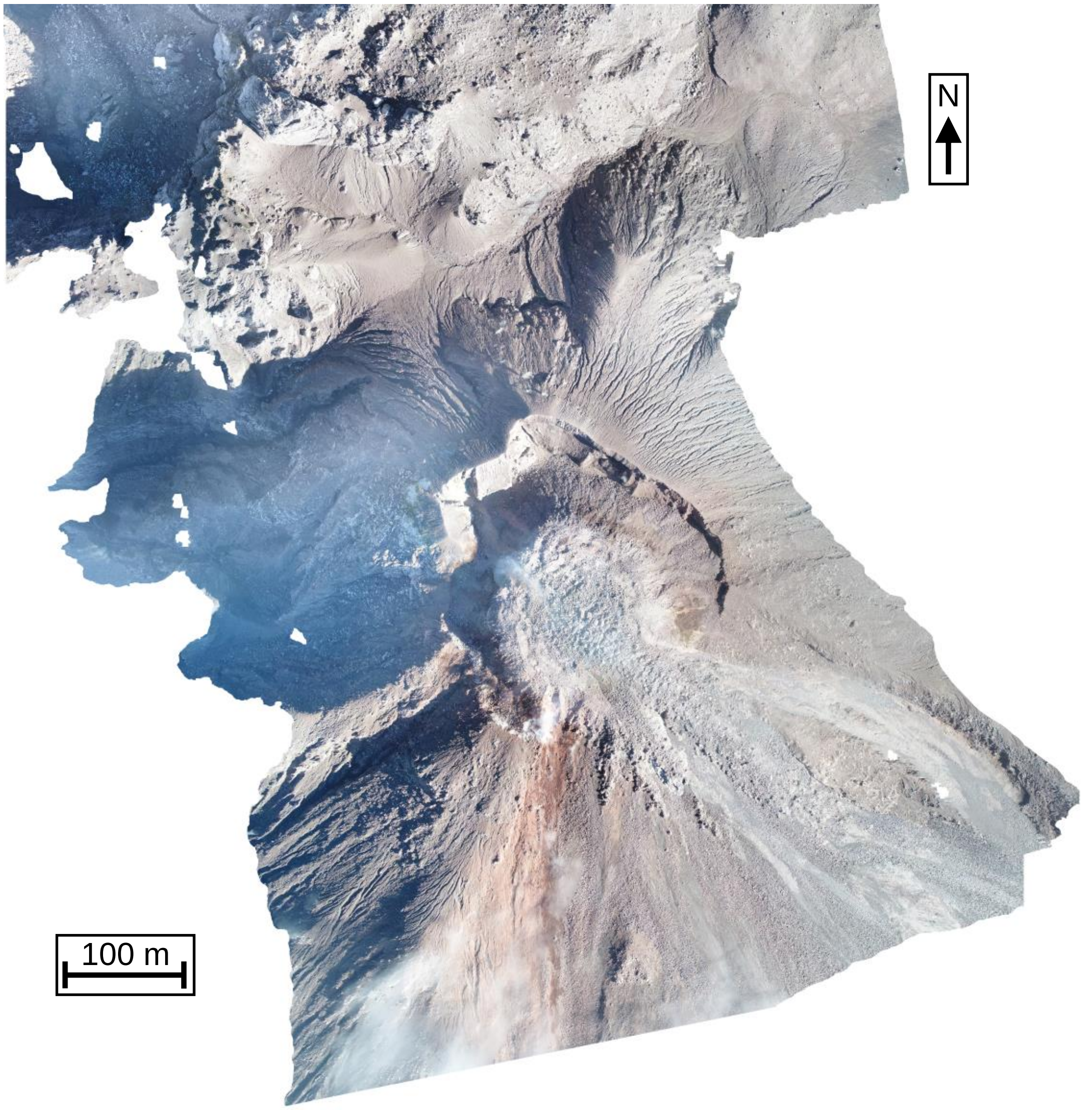

Supplement: Supplementary file 1 — Supplementary Information. [file 41598_2020_65386_MOESM1_ESM.pdf]
