# Supplementary figures and images for: UAS-based tracking of the Santiaguito Lava Dome, Guatemala
Source: Sci Rep. 2020 May 25;10:8644. doi: 10.1038/s41598-020-65386-2 (PMC7248112; doi:10.1038/s41598-020-65386-2)

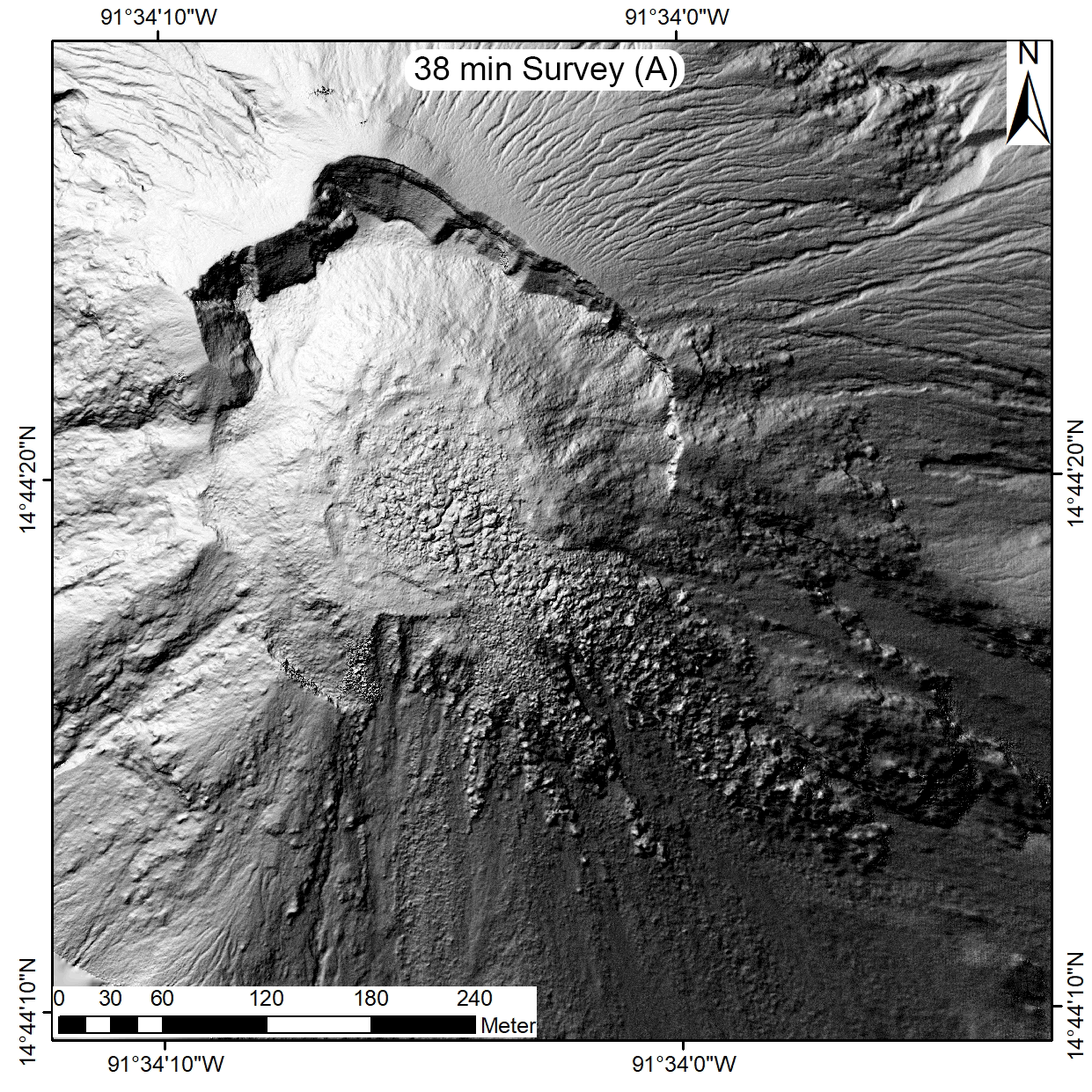

Supplement: Supplementary file 2 — Supplementary Figure S9. [file 41598_2020_65386_MOESM2_ESM.gif]

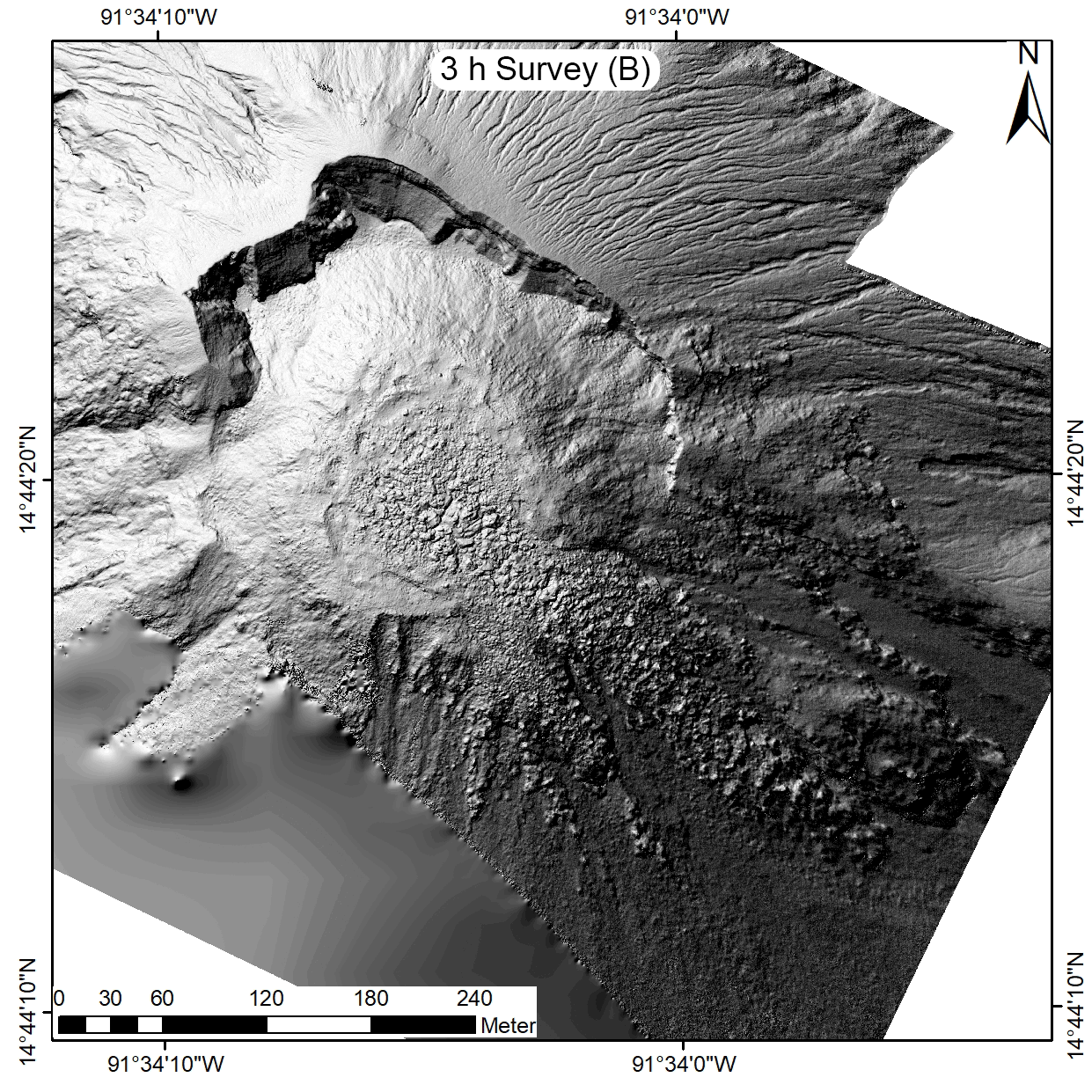

Supplement: Supplementary file 3 — Supplementary Figure S10. [file 41598_2020_65386_MOESM3_ESM.gif]

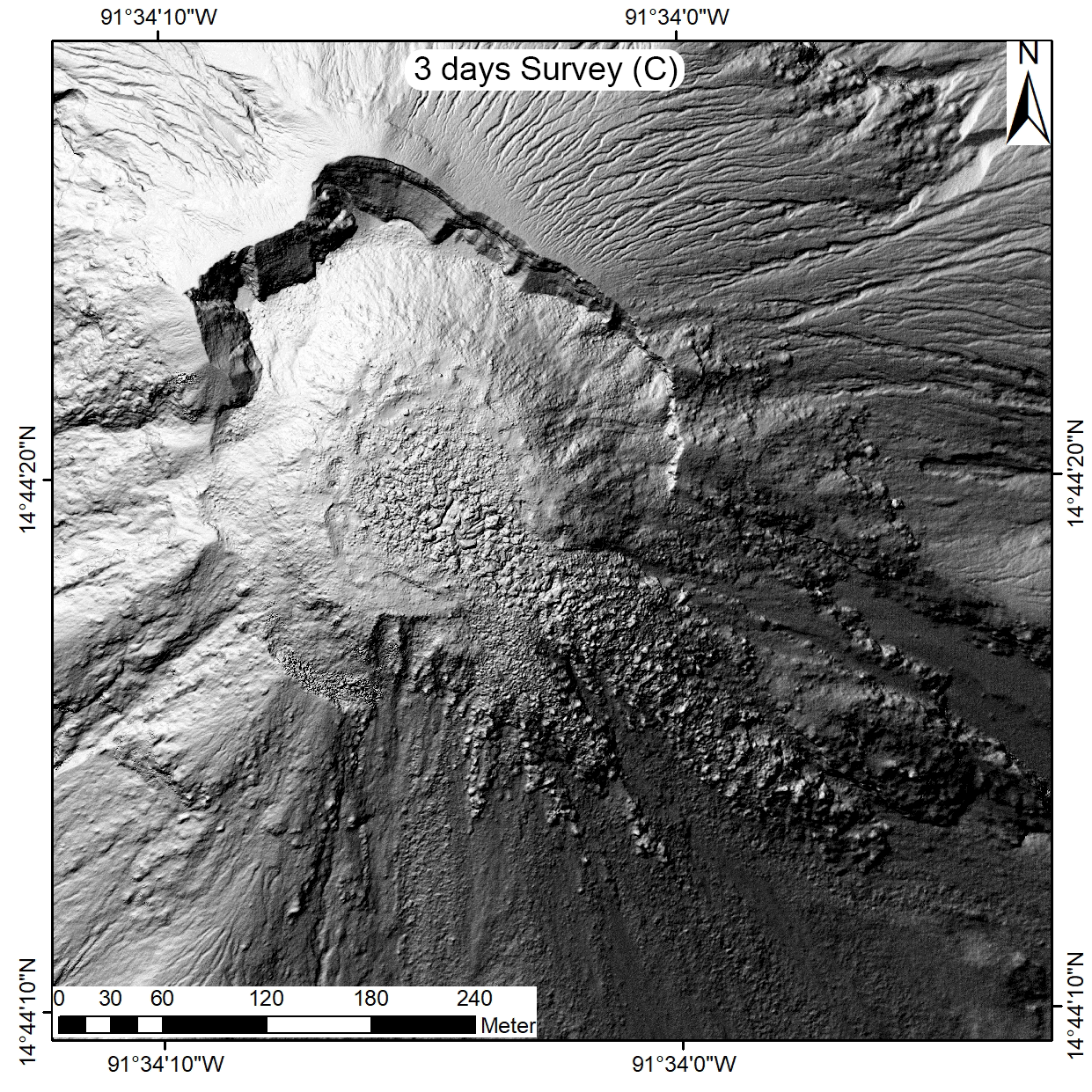

Supplement: Supplementary file 4 — Supplementary Figure S11. [file 41598_2020_65386_MOESM4_ESM.gif]
